# Safety and Efficacy of Mepolizumab in Patients with Eosinophilic Granulomatosis with Polyangiitis

**DOI:** 10.1155/2019/4376380

**Published:** 2019-03-03

**Authors:** Ravi Ranjan Pradhan, Gaurav Nepal, Shobha Mandal

**Affiliations:** ^1^Tribhuvan University Institute of Medicine, Kathmandu, Nepal; ^2^Regional Neurological Associates, New York, USA

## Abstract

Eosinophilic granulomatosis with polyangiitis (EGPA) is a rare form of vasculitis disorder which involves multiple organ systems and is characterized by asthma, pulmonary infiltrates, sinusitis, neuropathy, and peripheral eosinophilia. It also has an effect on the heart, skin, kidneys, and gastrointestinal tract. Interlukin-5 (IL-5) is involved in maturation and activation of eosinophil, the production of which is increased in the EGPA. Treatments of EGPA are limited to systemic corticosteroids and immunomodulators. These drugs are associated with significant side effects. Besides this, the response of patients to these drugs may be disappointing. Frequent relapses, the need for long-term medium-to-high-dose glucocorticoid therapy, and failure to achieve remission are not uncommon findings. There is a need for noble agents that could reduce frequent relapses and the dose of systemic glucocorticoids and maintain a sustained remission without significant side effects. Mepolizumab is IL-5 antagonist and may have value in treating patients with EGPA. Therefore, we did a systematic review to evaluate the efficacy and safety of mepolizumab in patients with EGPA.

## 1. Introduction

Eosinophilic granulomatosis with polyangiitis (EGPA), which was previously recognized as Churg–Strauss syndrome (CSS), was first described in 1951 by Churg and Strauss. It is a small-vessel necrotizing vasculitis characterized by multisystemic manifestations like asthma, lung infiltrations, extravascular necrotizing granuloma, and hypereosinophilia [1]. The most commonly involved organ is the lung, followed by the skin. EGPA can virtually affect any organ systems, including cardiovascular, gastrointestinal, renal, and central nervous system [2]. Vasculitis of extrapulmonary organs is largely responsible for the morbidity and mortality in patients with EGPA.

EGPA typically occurs in several phases. The prodromal phase is characterized by asthma and/or allergic rhinitis, which usually begins when the individual is in their second to third decade of life. The eosinophilic infiltration phase is characterized by peripheral eosinophilia and eosinophilic tissue infiltration of different organs. The third phase is the vasculitic phase and it is associated with constitutional signs and symptoms like fever, malaise, fatigue, and weight loss.

Treatments of EGPA are limited to systemic corticosteroids and immunomodulators. These drugs are associated with significant side effects. Despite treatment, the response to disease is limited. Frequent relapses, need for long-term medium-to-high-dose glucocorticoid therapy, and failure to achieve remission are not uncommon findings.

The exact pathogenesis of EGPA is poorly understood. Antineutrophil cytoplasmic antibodies (ANCA) are detected in about 40 to 60 percent of patients with EGPA and are classified among the ANCA-positive vasculitides [3]. In addition, EGPA is characterized by several other abnormalities of immune function such as heightened Th2 and Th1 immunity (suggested by prominence of allergic features and pulmonary angiocentric granulomatosis, resp.) and altered humoral immunity (suggested by increased serum IgE level) [4, 5]. Studies suggest a direct pathogenic effect of eosinophilic infiltration in the different tissues [4, 6, 7]. Interlukin-5 (IL-5) mediates proliferation, maturation, differentiation, tissue survival, and activation of eosinophil [8, 9]. Levels of IL-5 are increased in patients with EGPA and might correlate with disease activity [5]. Therefore, neutralization of IL-5 offers a rational therapeutic approach for managing a case of EGPA. Mepolizumab is an anti-IL-5 monoclonal antibody that binds to IL-5 and prevents the interaction of IL-5 with its receptor on the surface of eosinophil. Mepolizumab is found to be effective in reducing peripheral eosinophil counts in different hypereosinophilic syndrome [10–12]. In this review, we reviewed the current evidence for the efficacy and safety of mepolizumab in patients with EGPA.

## 2. Methods

### 2.1. Search Strategy

**Figure 1:**
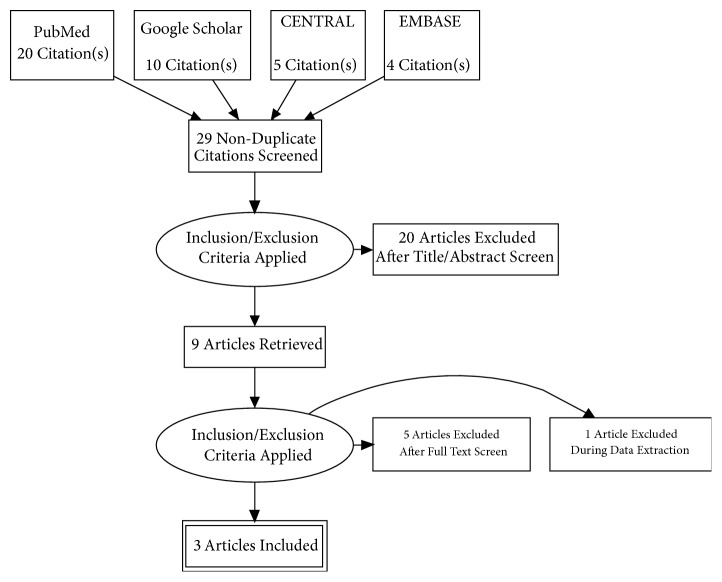
PRISMA diagram detailing the study identification and selection process.

The PRISMA statement for reporting systematic reviews recommended by the Cochrane Collaboration was followed for conducting this systematic review [Figure 1]. PubMed, Google Scholar, CENTRAL, and EMBASE were searched for peer-reviewed research published between July 2005 and July 2018. Databases were searched using the search terms under two search themes and combined using the Boolean operator “AND”. For the theme “Mepolizumab”, we used the following text words: mepolizumab, IL-5 antagonist, and monoclonal antibody. For the theme “Eosinophilic granulomatosis with polyangiitis”, we used the following text words: Eosinophilic granulomatosis with polyangiitis, Churg-Strauss Syndrome, EGPA, and CSS [13].

### 2.2. Selection Criteria

Studies published in the English language were included in the review if they aimed to assess efficacy and safety of mepolizumab in patients with EGPA. Studies that aimed to assess efficacy and safety of mepolizumab in disease conditions other than EGPA, like asthma, hypereosinophilic syndrome, and eosinophilic esophagitis, were excluded. In addition, case reports, case series, editorials, and correspondences were also excluded [13]. Diagram detailing the study identification and selection process is given in Figure 1.

### 2.3. Data Abstraction

The authors (RRP and GN) independently screened the articles based on the inclusion and exclusion criteria. Full texts were obtained for articles that met inclusion criteria. The authors developed a data abstraction spreadsheet using Microsoft Excel version 2013 (Microsoft Corp., Redmond, WA, USA) and included the following information: author, year of publication, journal, country where the study was done, study design, sample size, baseline characteristics of the patients, dose, frequency, and the route of administration of the drug, efficacy in the terms of period of remission, relapse, and reduced dose of steroid, and the safety of the drug. Any discrepancies were solved by consultation with a third author (SM) [13].

## 3. Results and Discussion

### 3.1. Study Characteristics

The study characteristics are represented in Table 1. All the three articles included in this review were of good quality, considering the presence of clear objectives, a clearly mentioned study design, and clearly described statistical analysis. Three trials were included in this systematic review, with a total of 153 subjects. All the studies have used their own exclusion and inclusion criteria. Wechsler et al. [14] and Moosig et al. [15] have used separate criteria for remission, but Kim et al. [16] have not included criteria for remission in their study.

### 3.2. Patient Characteristics

The patient characteristics of the study are shown in Table 2. The mean age of patients in Wechsler et al.'s work was 49 years and 48 years for case and control, respectively. Similarly, the mean age of the patients included in Kim et al.'s work and Moosig et al.'s work was 45 years and 62 years, respectively. Mean forced expiratory volume in one second (FEV_1_) and Birmingham Vasculitis Activity Score (BVAS) were similar in Kim et al.'s and Moosig et al.'s studies.

### 3.3. Comparison of Treatments

The studies conducted by Kim et al. and Moosig et al. used mepolizumab 750 mg intravenous infusion once in every four to six weeks. Wechsler et al. examined a dose of mepolizumab as 300 mg subcutaneously every 4 weeks. In all the three studies, glucocorticoid was tapered gradually according to a standardized recommended tapering schedule few weeks after starting mepolizumab.

### 3.4. Comparison of Outcomes

The outcomes of all studies were remission, relapse, an average daily dose of prednisolone or prednisone, and the safety of mepolizumab. However, remission was not included in the study of Kim et al.

### 3.5. Efficacy and Safety

Wechsler et al. established that treatment with mepolizumab led to significantly more accrued weeks of remission than placebo (28% versus 3% of the participants had ≥ 24 weeks of accrued remission; odds ratio, 5.91; 95% confidence interval [CI], 2.68 to 13.03; p < 0.001) and a significantly higher percentage of participants were in remission at both weeks 36 and 48 compared to placebo (32% versus 3%; odds ratio, 16.74; 95% CI, 3.61 to 77.56; p < 0.001). Overall, 44% of mepolizumab-treated subjects were able to taper prednisolone or prednisone to 4 milligram (mg) or less per day, compared with 7% of subjects taking placebo. The time to first relapse over 52-week period was longer in mepolizumab than in placebo (56% versus 82%; hazard ratio, 0.32; 95% CI, 0.21 to 0.50; p < 0.001). In the same study, most commonly reported adverse events were headache (32% in mepolizumab group and 18% in placebo group), nasopharyngitis (18% versus 24%), arthralgia (22% versus18%), sinusitis (21% versus 16%), upper respiratory tract infection (21% versus 16%), exacerbation or worsening of asthma (3% versus 6%), and local injection-site reactions (similar in the two groups). One patient in mepolizumab group died from cardiac arrest during this study; however, this participant had a prior history of coronary artery disease [14].

In the study performed by Kim et al., there was significantly lower exacerbation rate during the treatment period (0.14 events per week, two events during a 14-week period) compared to the nontreatment period (0.69 events per week, 18 events over a 26-week period). In this study, they found that mepolizumab effectively served as a corticosteroid-sparing therapy. The mean dose at baseline was 12.9 milligrams (mg) per day, which was reduced to 4.6 mg per day after 12 weeks of therapy (study week 16), that is, 64% reduction in corticosteroid dose (p=0.0001) after 4 doses of mepolizumab. As per this study, mepolizumab was safe to use, and there were no severe adverse events noted during the study period. The common adverse events were mild transient headache (n= 3), mild pruritus (n=1), and loose stool (n=1). In the same study, participants experienced wheeze or cough (n=4), sore throat (n=4), nausea or abdominal discomfort (n=3), sinusitis (n=6), and arthritis (n=1) during the trial. However, these adverse events were not due to mepolizumab; rather they were presumably related to corticosteroid tapering, signs of EGPA activity, or both [16].

In a similar study conducted by Moosig et al., out of 10 patients, eight reached remission, one had BVAS of zero but did not achieve glucocorticoid dose less than 7.5 mg/day, and one reached remission but was excluded because of nonadherence. There was no relapse noted with mepolizumab therapy. The daily dose of glucocorticoid was reduced significantly at week 32 (median, 19 mg at baseline to 4 mg at week 32; p=0.006). Mepolizumab was well tolerated and the most common adverse events associated with mepolizumab therapy were eczema, edema, swelling of left hand, urinary tract infection, dentalgia, abdominal pain, wound infection, otitis media, bronchitis, herpes zoster, and herpes simplex. Severe adverse events like anaphylaxis (n=1), norovirus infection (n=1), cerebral micro embolism (n=1), and de Quervain thyroiditis (n=1) were noted. However, these serious adverse events were probably unrelated to mepolizumab therapy [15].

The present work is, to the best of our knowledge, the first systematic review of the latest three trials, allowing the direct comparison of efficacy and safety of mepolizumab in patients with EGPA.

### 3.6. Conclusion of Included Studies

Based on the studies analyzed, we found that mepolizumab allows substantial corticosteroid tapering at the cost of maintaining clinical stability. In all the studies, there was a significant decrease in mean corticosteroid dose few weeks after start of therapy with mepolizumab. We also found a significantly higher proportion of remission and a lower rate of relapse with mepolizumab therapy. Mepolizumab does not only control the asthma symptoms but also has an effect on systemic vasculitis manifestations as suggested by decreased BVAS in all the three studies. The safety of mepolizumab is well established from the three studies, except for few minor side effects. The serious adverse events also occurred during the trials; however, those events were probably unrelated to mepolizumab therapy. In all the three studies, the pattern of exacerbation, clinical symptoms, and dose of corticosteroid improved while patients were under mepolizumab therapy but worsened after it was withdrawn. Given the substantial adverse effects of long-term systemic corticosteroid, such as weight gain, impaired blood sugar, iatrogenic Cushing syndrome, thinning of skin, osteoporosis, adrenal suppression, and increased risk of infection, mepolizumab could establish itself as a noble agent in treating patients with EGPA.

### 3.7. Comparison with Other Studies

A meta-analysis of randomized placebo-controlled trials of mepolizumab in patients with eosinophilic asthma found significantly decreased exacerbation risk compared to placebo (OR, 0.30; 95%CI, 0.13 to 0.67; p=* *0.004) and a significant improvement in the scores on the Asthma Quality of Life Questionnaire (AQLQ) (mean difference, 0.26; 95% CI, 0.03 to 0.49; p* *=* *0.03). The same study found that mepolizumab was well tolerated. Some serious adverse events reported such as cerebrovascular disorder, asthma exacerbation, and gastrointestinal disturbance were not considered by the investigators to be related to study medication. The common adverse events were headache, chest pain, facial flushing, erectile or ejaculatory dysfunction, rash, conjunctivitis, fatigue, upper respiratory tract infection, rhinitis, bronchitis, sinusitis, viral infection, injury, nausea, and pharyngitis [17].

A study conducted by Nair et al. in patients with prednisone-dependent asthma with sputum eosinophilia found that the rate of exacerbation was significantly lower in mepolizumab group compared to placebo group. Patients who received mepolizumab were able to reduce their prednisone dose by a mean (±SD) of 83.8±33.4% of their maximum possible dose, as compared with 47.7±40.5% in the placebo group (p=0.04). The use of mepolizumab was associated with a significant decrease in the number of sputum and blood eosinophils. There were no serious adverse events [18].

A randomized controlled trial done by Rothenberg et al. in patients with negative* FIP1L1–PDGFRA* fusion gene hypereosinophilic syndrome with mepolizumab discovered that mepolizumab treatment enabled clinically significant reductions in corticosteroid dose and often corticosteroid discontinuation [19].

Studies have shown that mepolizumab is associated with marked decreases in peripheral blood and esophageal eosinophilia in patients with eosinophilic esophagitis and improved clinical outcomes [20, 21].

### 3.8. Justification of Use

Treatment of EGPA remains a challenge for physicians because the current available therapies, corticosteroids and immunomodulators, do not always control symptoms and are often associated with significant morbidity and relapses. The long-term use of high-dose corticosteroid is associated with potential adverse effects like impaired blood sugar, increased risk of infection, Cushing syndrome, glaucoma, weight gain, and adrenal suppression. So it is necessary to find an alternative to corticosteroids and immunomodulators. Mepolizumab is a potential alternative that binds to IL-5 and prevents its interaction with its receptor on the eosinophil surface. Besides EGPA, mepolizumab is also found to be effective in other hypereosinophilic syndromes. The safety of mepolizumab is well established from different studies.

### 3.9. Limitations of the Review

The result of our systematic review should be considered with caution, owing to limited number of available studies and subjects. The sample size was small to reach a convincing conclusion. Secondly, the drug dose and treatment duration differing in the trials involved in our review made it difficult to determine the optimal dose of mepolizumab which would be mostly appropriate for patients with EGPA.

## 4. Conclusions

To conclude, this review has found that mepolizumab is efficacious and safer to use in patients with EGPA. It has an effect on asthma symptoms as well as systemic vasculitis manifestations. It might improve the rate of remission, decreases relapse rate, and allow for reduced glucocorticoid use, at cost of any serious adverse drug effects.

## Figures and Tables

**Table 1:** Key methodological characteristics of selected studies.

Author	Year	Country	Journal	Sample size	Study design	Inclusion criteria	Exclusion criteria	Primary end point	Mepolizumab dose
Wechsler et al. [14]	2017	USA	NEJM	68 versus 68	RCT	Age 18 years and above Diagnosis of relapsing or refractory EGPA at least 6 months previously and had been taking stable dose of prednisolone or prednisone (≥7.5 to ≤50.0 mg per day, with or without additional immunosuppressive therapy) for at least 4 weeks before the baseline visit	Granulomatosis with polyangiitis or microscopic polyangiitis Organ-threatening or life-threatening eosinophilic granulomatosis with polyangiitis within 3 months before screening	*∗*Accrued weeks of remission over a 52-week period Proportion of participants in remission at both week 36 and week 48	300 mg s.c. every 4 weeks

Kim et al. [16]	2010	USA	Journal of Allergy and Clinical Immunology	7	Open-label pilot study	A diagnosis of CSS as defined by the American College of Rheumatology classification criteria. Patient to be maintained on a stable dose of at least 10 mg of prednisone daily (or equivalent) before enrollment. Subjects receiving adjunct therapies, such as cyclophosphamide, azathioprine, or methotrexate, were required to maintain a stable dose so that medication withdrawal would not obfuscate potential drug benefits.	Patients with non-CSS hypereosinophilic syndromes, Wegener granulomatosis, malignancy, or parasitic disease. Pregnant or nursing female patients. Female subjects with child-bearing potential.	Whether mepolizumab safely decreased CSS disease activity and permitted scheduled tapering of systemic Corticosteroids. Lowest prednisone dose achieved at the end of the treatment phase	750 mg i.v. infusion every four to six weeks

Moosig et al. [15]	2011	Germany	Annals of Internal Medicine	10	Uncontrolled trial	Patients with active refractory or relapsing active Churg–Strauss syndrome, defined by a Birmingham Vasculitis Activity Score (BVAS) greater than 3 despite treatment with immunosuppressants plus glucocorticoids at a dosage of 12.5 mg/d or higher.	NA	Remission at week 32, as defined by recommendations from the European League Against Rheumatism of a BVAS of 0 and a glucocorticoid dosage of less than 7.5 mg/d.	750 mg i.v. infusion every 4 weeks

*∗*Remission was defined as Birmingham Vasculitis Activity Score (BVAS) of zero and the receipt of prednisolone or prednisone at a dose of 4 mg or less per day over the 52-week period.

**Table 2:** Baseline characteristics of patients included in selected studies.

Study	Mean age (years)	Mean FEV_1_ (percentage)	Eosinophil count	Mean corticosteroid dose (mg)	BVAS	CRP (mg/L)	ESR (mm/hr)
Wechsler et al. [14]	CASE: 49 CONTROL: 48	NA	AEC per cubic millimeter CASE: 177 Control: 172	12 11	NA	NA	NA

Kim et al. [16]	45	76	3.4 %	12.9	6.9	3.9	7

Moosig et al. [15]	62	76.24	AEC per cubic millimeter: 539	18	6.9	0.68	22.7

## Abbreviations

AEC: Absolute eosinophil count
AQLQ: Asthma Quality of Life Questionnaire
BVAS: Birmingham Vasculitis Activity Score
CI: Confidence interval
CRP: C-reactive protein
CSS: Churg–Straus syndrome
EGPA: Eosinophilic granulomatosis with polyangiitis
ESR: Erythrocyte sedimentation rate
FEV: Forced expiratory volume
i.v.: Intravenous
IL-5: Interlukin-5
NA: Not available
NEJM: New England Journal of Medicine
RCT: Randomized Controlled Trial
s.c.: Subcutaneous
USA: United States of America.
